# Filling the Permanent Teeth of Children

**Published:** 1885-02

**Authors:** C. M. Marshall


ARTICLE VI.

### FILLING PERMANENT TEETH OF CHILDREN.

BY C. M. MARSHALL.

Having had occasion, during the past few years, to extract many teeth for children between the ages of eight and fifteen, that han been filled with cohesive gold, and as such cases continue to present themselves to me, I wish to earnestly protest against such practice. I consider it pernicious.

1st. Because gold is not the best material we have for filling such teeth.

2d. Because a filling that will not save a tooth, serves usually to mask the real condition until pulp irritation, and frequently inflammation, ensues.

3d. Because we have filling materials that will arrest decay in these teeth, and preserve them until maturity is reached, when they will be sufficiently hardened to receive gold as permanent work.

To return to my first statement: Before maturity, many teeth are so soft that the pressure necessary to impact cohesive gold solidly in apposition to the walls of a cavity, will, in a great majority of cases, cause fractures of of the edges of the cavity. Though they may sometimes be infinitesimal, they will yield to the action of the fluids of the mouth, and further caries follow.

I moreover claim that gold is not the best filling material for such teeth, inasmuch as it requires such great endurance that a child will frequently leave your chair wishing he had not a "tooth in his head," mentally vowing he will never have another filled. Thus children in their tender years conceive an antipathy for you and your practice, which only grows with time.

In my second statement I occupy ground familiar to all. If not, let the operator who has been pursuing the course herein condemned for at least three years, come to the front and teach us, and tell us he never finds anything wrong around his gold fillings in such teeth. I grant many are saved, but are we meeting the demands rightfully made upon us if we lose any, when we have charge of them at the time caries first appear, and the proper hygienic laws are obeyed? Why should one of four incisors, with caries of similar extent, be lost, after an equal amount of care has been bestowed upon filling each? If the nonsense of incompatibility is manifest in the lost tooth, why does it not exhibit itself in the others? If the mystical electrolysis does not disintegrate three, why should the fourth yield to it?

I claim for third position, that children's permanent teeth, though sometimes deficient in lime salts, can be preserved if the proper course is judiciously pursued, and in doing this I do not pretent to offer anything new to the profession, but to reiterate truths already spoken, which have proven by practice worthy of repetition. I simply add my protest against the old methods, trusting that if there is one practitioner who has not before, he may now, investigate this matter, and become better prepared to meet the just demands imposed upon him, thus honoring his profession more highly, and adorning his personal character, by increasing the gratitude and confidence of his patrons.

I recommend this practice: Fill teeth which are exceedingly deficient in stuctural constituents with either oxyphosphates or oxy-chlorides. These fillings are only intended to serve a period of six months or two years, the time depending much upon the chemical reaction of the fluids of the mouth. If, when they are partly solved, we do not find a great improvement in the texture of the teeth, we should refill them with the same material, and repeat it until there is sufficient integrity of tooth structure established to maintain more permanent work. Where we find these teeth fairly supplied with lime salts, a high standard of amalgam may be used, especially for bi-cuspids and molars, which will serve much the same purpose, requiring less frequent renewal, and possibly none at all. By this means we can bridge over the most dangerous period of carious permanent teeth, and finally fill them with cohesive gold.—*Southern Dental Journal.*

---

